# Randomised clinical trial: effect of low-FODMAP rye bread versus regular rye bread on the intestinal microbiota of irritable bowel syndrome patients: association with individual symptom variation

**DOI:** 10.1186/s40795-019-0278-7

**Published:** 2019-03-06

**Authors:** Reijo Laatikainen, Jonna Jalanka, Jussi Loponen, Sanna-Maria Hongisto, Markku Hillilä, Jari Koskenpato, Riitta Korpela, Anne Salonen

**Affiliations:** 1 Faculty of Medicine, Pharmacology, Medical Nutrition Physiology, University of Helsinki, Helsinki, Finland; 2 Aava Medical Centre, Helsinki, Finland; 3 Human Microbiome Research Program, Faculty of Medicine, University of Helsinki, Helsinki, Finland; 4 Fazer Group, Vantaa, Finland; 5 Clinic of Gastroenterology, University of Helsinki and Helsinki University Hospital, Helsinki, Finland; 6 Booston Oy Ltd, Viikinkaari 6, 00790 Helsinki, Finland

**Keywords:** Intestinal microbiota, FODMAP, Irritable bowel syndrome, IBS, Rye, Diet, Bifidobacteria, *Blautia*, Symptoms, Responder

## Abstract

**Background:**

A low intake of Fermentable, Oligo-, Di-, Mono-saccharides and Polyols (FODMAPs) is effective in the symptom control of irritable bowel syndrome (IBS) patients but may exert negative effects on the intestinal microbiota. The microbial effects of increasing regular or non-FODMAP fibre sources are largely unknown. Furthermore, it is not known if the baseline microbiota composition is associated with individual symptom control during the consumption of different rye products in IBS patients. Our objective was to evaluate whether increased consumption of low-FODMAP rye bread or regular rye bread for 4 weeks would alter the intestinal microbiota composition of IBS patients following their habitual diet, and whether these changes associate to symptoms and/or the baseline microbiota.

**Methods:**

The study was conducted as a randomized double blind controlled cross-over study (*n* = 50). Microbiota was analysed by 16S rRNA gene sequencing and associated with gastrointestinal symptoms. Both microbial changes and their associations to symptoms were secondary outcomes.

**Results:**

The consumption of the test breads did not alter microbiota diversity. Compared to baseline, consumption of the low FODMAP rye bread decreased the abundance of *Bacteroides*, *Flavonifractor*, *Holdemania, Parasutterella* and *Klebsiella* and showed a trend towards increased bifidobacteria, whereas the regular rye bread decreased the abundance of *Flavonifractor.* When comparing between the two test breads, *Klebsiella* was decreased after low-FODMAP rye bread intake*.* Patients whose symptoms decreased during the low-FODMAP rye bread displayed more *Blautia* and less *Barnesiella* at baseline.

**Conclusions:**

Consumption of low-FODMAP rye bread had modest, potentially beneficial effects on patients’ microbiota while increasing their intake of fibre substantially. The baseline microbiota composition was associated with the variable degrees of symptom relief experienced by the patients. Consumption of a low-FODMAP rye bread might be one way to increase dietary fibre intake and improve the mild dysbiosis often observed among patients with IBS.

**Trial registration:**

ClinicalTrials.gov: NCT02161120. Retrospectively registered 11 June 2014.

**Electronic supplementary material:**

The online version of this article (10.1186/s40795-019-0278-7) contains supplementary material, which is available to authorized users.

## Background

Irritable bowel syndrome (IBS) is the most common disorder diagnosed by gastroenterologists. On average, it affects 11% of the adult population in the developed countries [1]. Adoption of a low-FODMAP diet, i.e. a dietary approach restricting intake of poorly absorbable, fermentable carbohydrates, has achieved acceptance in the treatment of IBS both among gastroenterologists and dieticians [2] because its use significantly reduces the symptoms of IBS [3].

One disadvantage linked with a low-FODMAP diet is its potential negative effect on the intestinal microbiota. Especially the reduction of beneficial bifidobacteria and also the reduced relative abundance of Akkermansia *muciniphila, Faecalibacterium prausnitzii* and *Lactobacillus* as well as an increased tendency towards dysbiosis have been reported [4–7]. The clinical importance of these theoretically unfavourable microbiota alterations after adoption of a low-FODMAP diet is not fully understood. Nonetheless, it seems reasonable to postulate that sustaining a high relative abundance of these beneficial microbes would be advantageous also in IBS patients to prevent long-term adverse effects on gut and systemic health.

Whole grains are considered as healthy foods, mainly due to their high fibre content [8]. A high consumption of rye has been associated with a reduced risk of death in men [9]. By consuming rye, an individual increases his/her intake of cereal fibre and this has been linked to a lower risk of colorectal cancer and cardiovascular disease [10, 11]. On the other hand, grains are often considered as triggers of IBS symptoms, e.g. rye is high in FODMAPs, thus limiting the consumption of rye products by IBS patients [2].

We have previously shown that low-FODMAP rye is better tolerated than the regular rye bread in IBS patients [12]. The high consumption of low-FODMAP rye bread allowed IBS patients to increase their fibre intake by 7 g/day without compromising the symptom control.

These findings and the lack of any prior studies on the effect of rye on the microbiota of IBS patients prompted us to investigate whether a substantial increase in the consumption of rye bread would induce changes in the microbiota of IBS patients who otherwise followed their habitual diet, and whether these changes would differ between a low-FODMAP rye bread and regular rye bread. We have previously shown that the baseline intestinal microbiota composition may be related to the host’s physiological response to whole grains and other dietary fibres [13]. In addition, recent studies in IBS patients have provided evidence that the resident microbiota influences the efficacy of a low-FODMAP diet in symptom control [6, 14, 15]. Here, we examined whether the microbiota of patients with clear symptom resolution following the intake of the low-FODMAP bread, or those in whom symptoms worsened after consuming the regular rye bread, differed from the other participants in the trial. In summary, we compared the microbial effects of low-FODMAP-rye bread and the regular rye bread in a randomized, double-blinded, crossover study in patients with IBS and correlated the microbiota profiles with IBS symptoms, and searched for potential microbiota signatures which would characterize those patients whose symptom control was profoundly altered following the intake of either of the rye breads.

## Materials and methods

### Subjects and study design

**Fig. 1:**
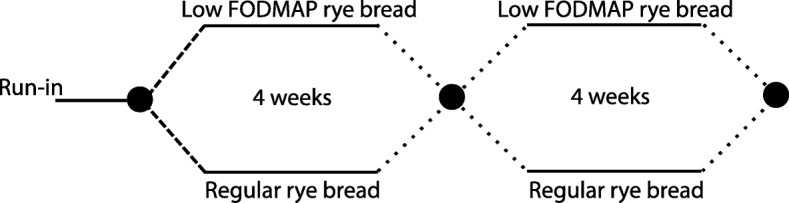
Study design

A total of 87 subjects with IBS were recruited into the study with 14 withdrawing from the study prematurely. Only those subjects for whom fecal samples were successfully collected and analysed at all three time points were utilized in this study (*n* = 50). The subjects, study design, measurement of symptoms, measurement of colonic fermentation (breath hydrogen test), withdrawals, flow chart, diets and quality of breads have been described in detail in the report of the study’s primary outcomes [12]. In short, this study (ClinicalTrials.gov: NCT02161120) was a randomised, double blind, 2 × 2 cross-over trial with a 1 week run-in period when participants adhered to their habitual diet, followed by two 4-week treatment periods during which the patients consumed either the low-FODMAP rye bread or a regular rye bread, with a washout period of ≥4 weeks between these two periods (Fig. 1). The study protocol was approved by the ethics committee of the Hospital District of Helsinki and Uusimaa and all participants gave written consent. Changes in microbiota and their associations to symptoms as described in this report were secondary outcomes of the clinical trial.

### Study breads

The breads were developed and supplied by Fazer Bakeries (Vantaa, Finland). The breads had a similar appearance, taste and were packaged in identical transparent plastic bags. The control bread was prepared using traditional rye sourdough whereas the low-FODMAP rye bread was prepared using a specific sourdough system which resulted in a rye bread with a clearly reduced FODMAP (fructan and mannitol) content (12). The low-FODMAP rye bread contained FODMAPs 0.4 g/100 g whereas regular bread contained 1.4 g of FODMAP /100 g. The participants were requested to consume 7–8 slices of rye bread per day depending on individual energy needs during the 2nd, 3rd and 4th week; this led to a difference of 2.1–2.4 g in their intake of FODMAPs/day. During the first week of the treatment periods, the targeted rye bread dose was halved, i.e. 3.5–4 slices. Further details can be found in the previous publication (12) and in Table 1.

**Table 1:** Nutritional composition of the breads. Reproduced from [12] with Creative Commons Attribution permission

	Low-FODMAP rye bread */100 g	Regular rye bread*/100 g
Energy, kJ (kcal)	1024 (245)	1033 (247)
Protein, g	9.2	9.3
Fat, g	1.3	1.2
Carbohydrates, g	43.6	44.0
Dietary Fibre, g	10.2	10.5
Sodium, g	0.4	0.4
Fructans, g	0.3	1.1
Mannitol, g	0.1	0.3
LMWDF, g	1.9	2.6
Insoluble HMWDF, g	5.9	5.4
Soluble HMWDF, g	2.5	2.4
Resistant starch, g	0.9	0.8

*LMWDF* Low Molecular Weight Dietary Fibre, *HMWDF* High Molecular Weight Dietary Fibre

*) Participants were supplied with and instructed to consume 3.5–4 slices (105–120 g) of each bread/day during the trial’s first week and 7–8 slices (210–240 g) during weeks 2–4 of the study

### Fecal samples and DNA extraction

Fecal samples were collected at home at baseline and on the 2nd last day of each intervention arm. The samples were immediately stored at −20 °C and transported to the study centre for DNA extraction within 6 months. Bacterial DNA was extracted from approx. 125 mg of fecal matter using the Repeated Bead Beating (RBB) method [16] with the following modifications for automated DNA purification: After the beat beating steps, 400 μl of the cell lysate pooled from the two beat beating rounds was purified with the RSC Blood DNA kit AS1400 in a Promega Maxwell RCS instrument (Promega, Madison, WI, USA). DNA was quantified using Quanti-iT™ Pico Green dsDNA Assay (Invitrogen, San Diego, CA, USA).

### Microbiota analysis

Sample preparation for Illumina MiSeq paired-end sequencing of the hypervariable V3-V4 regions of the 16S rRNA gene was performed according to the protocol recommended by Illumina with a few modifications as previously described [17].

### Sequencing data preprocessing, analysis and statistics

The pre-processing of the sequencing reads, their taxonomic annotation and statistical analysis were performed in R using the package mare (Microbiota Analysis in R Easily) [18] as previously described [19] with the following modifications: Reads < 0.001% were removed as potentially erroneous and taxonomic mapping was done using Silva database, which was pre-filtered to include only gut-associated taxa. Samples with less than reads 3000 were discarded. The microbiota differences between the intervention and responder groups were analysed using generalized linear models with negative binomial distribution for bacterial genera detected in > 10% of the samples. Two models were fitted for each taxon: one with all data points and one with only the data points with non-zero values. The effect of variable read count was controlled by using the read count as an offset in all statistical models. Age, BMI and treatment order were used as confounders in all models with subject as a random factor. Gender was not associated to microbiota variation (permutational ANOVA r = 1%, *p* > 0.5) and was not used as a confounder. All *p*-values obtained from these models were adjusted for multiple testing using the Benjamini-Hochberg method and reported as P.adjust. In addition, the spread of the data was checked manually for all significant taxa obtained from the models to avoid reporting false positives. The β-diversity was estimated using Bray-Curtis dissimilarity as the distance measure and the contribution of different variables to microbiota variation was calculated using permutatonal ANOVA (vegan’s adonis function). The function “CorrelationMap” of the mare package was used to study the associations between bacteria and IBS symptoms. In the univariate data, a statistical difference was evaluated using t-test for two groups, and with ANOVA in combination with Tukey’s post-hoc test. For all tests, *p*-values < 0.05 were considered as statistically signifigant.

### Responder definition

We grouped the patients based on the change in their IBS-SSS and abdominal pain scores during the bread interventions. We focused on two responder groups, classified as follows: 1) A reduction in their IBS-SSS score by at least 50 points, as defined by Francis et al. [20], and/or symptoms of pain reduced by more than 10 mm in the VAS (mean of weekly measures) as compared to baseline during the low-FODMAP bread, or alternatively 2) an increase in their IBS-SSS score by at least 50 points and/or symptoms of pain increased by more than 10 mm at VAS (mean of weekly measures) compared to baseline during the regular rye bread. The symptom data were missing from one patient for each bread period, and hence 49 subjects were included in the responder analysis.

## Results

### Baseline characteristics

Table 1 depicts the nutritional composition of the breads including their FODMAP content and Table 2 summarizes the characteristics of the participants. Participant flow is described in Additional file 1: Figure S1. These patients can be classified as suffering from moderate IBS (mean IBS-SSS 235), as defined by Francis et al. [20].

**Table 2:** Baseline characteristics of the participants

Females, n (%)	47 (94)
Age (years), mean (range)	43.9 (21–64)
BMI (kg/m2), mean (range)	25.6 (21–64)
IBS symptom severity score, mean (SD; range)	235 (77;80–430)
IBS-M, mixed subgroup, n (%)	29 (62.5)
IBS-D, diarrhoeal subgroup, n (%) IBS-U, unspecified subgroup, n (%)	18 (32.5) 3 (5)

### Dietary intakes

The patients’ dietary intake is reported in Table 3. The mean intake of energy was 7981 kJ/day during baseline, 8171 kJ/day when they were eating the low-FODMAP rye bread and 8155 kJ/day when consuming the regular rye bread. There were no statistically significant differences in energy or macronutrient intake between the two interventions. Fibre intake increased by 7 g/day during the low-FODMAP rye bread period and by 8 g/day during the regular rye bread period as compared to the baseline period (*P* < 0.001 for both vs. baseline, *p* = 0.92 for between the test breads).

**Table 3:** Dietary intake during the baseline and during the consumption of the study breads

	Baseline *N* = 50	Low-FODMAP rye bread *N* = 44	Regular rye bread *N* = 48	*P* value*
Energy, kJ/d	7981 (±1992)	8171 (±2081)	8155 (±2466)	NS
Energy, kcal/d	1906 (±476)	1952 (±497)	1948 (±589)	NS
Carbohydrates, g/d	189 (±60)	199 (±60)	202 (±71)	NS
Protein, g/d	86 (±25)	86 (±25)	89 (±29)	NS
Fat, g/d	79 (±25)	78 (±26)	76 (±29)	NS
Total fibre, g/d	21 (±9)	28 (±9)	29 (±9)	<0.0001^1^

Results are given as mean (SD). *) Overall comparison between study periods using t-test analysis of variance. NS = No significant differences between any three periods. 1) both breads vs. baseline

### Community-level effects of the intervention on the intestinal microbiota

Our intestinal microbiota analysis was based on 10,554–66,363 (mean 35,757) high-quality MiSeq sequences per sample, representing 357 operational taxonomic units (OTUs) and 84 bacterial genera. The microbiota of the subjects at baseline consisted of Actinobacteria (mean 5.3%), Bacteroidetes (7.8%), Firmicutes (86.3%), Proteobacteria (0.3%) and Verrucomicrobia (0.3%). At the genus level, the most abundant bacteria were *Lachnospiraceae* Incertae Sedis (mean relative abundance 20.1%), *Bacteroides* (12.6%), *Faecalibacterium* (9.9%), *Blautia* (9.0%) and *Subdoligranulum* (7.8%).

We estimated the community dissimilarity (β-diversity) by performing a principal coordinates analysis (PCoA) using Bray-Curtis dissimilarity as the distance measure. There was no significant separation or distinct microbiota clustering attributable to the treatments or time points (*P* > 0.05). Permutational multivariate ANOVA, with Bray-Curtis dissimilarities, was used to assess the proportion of variation in the microbiota composition attributable to the time points (baseline, 1st and 2nd bread) and the treatments (low-FODMAP rye bread and regular rye bread). The time points and treatment order both explained only 1% of the microbial variation in these patients (*P* > 0.5, Additional file 2: Figure S2), indicating minor effects on the overall community structure. Similarly, α-diversity and/or richness were not affected by the intervention (*P* > 0.4 for all comparisons).

### Identification of specific bacterial taxa affected by the intervention

**Fig. 2:**
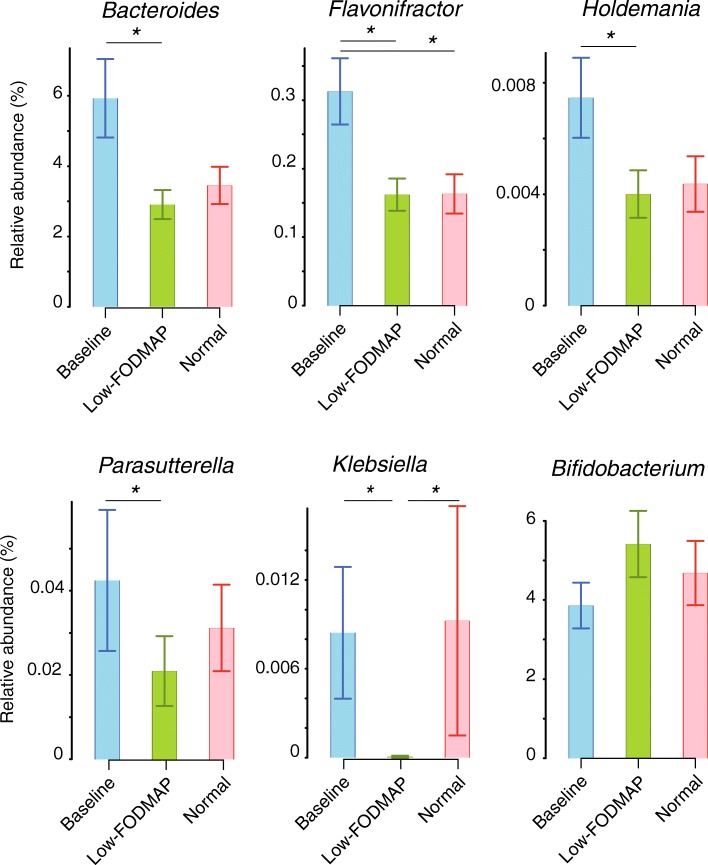
Relative abundance (± standard error) of the bacterial genera that differed between the baseline and low-FODMAP rye bread intervention, and abundance of bifidobacterial in all time points. Statistical significance between each treatment arm versus baseline was calculated with negative binomial models in the mare R package and is indicated with an asterisk (adjusted *p* < 0.05)

Next, we compared the relative abundance of bacterial genera between the treatments and the baseline. When comparing the two interventions, the abundance of *Klebsiella* was lower on the low-FODMAP rye bread period as compared to the regular rye bread (P.adjust = 0.048, fc = 14.8). This was the only statistical difference in bacterial taxa noted in the comparison between the breads. Both breads induced modest, partly overlapping effects on the microbiota. Altogether five bacterial taxa differed between the low-FODMAP rye bread and baseline (Fig. 2). During the low-FODMAP rye bread period, the abundances of *Bacteroides* (P.adjust = 0.03, fold change fc = 1.59), two Firmicute genera i.e. *Flavonifractor* (*Ruminococcaceae*, P.adjust = 0.06, fc = 1.55) and *Holdemania* (*Erysipelotrichaceae*, P.adjust = 0.08, fc = 1.84), and two Proteobacterial genera i.e. *Klebsiella* (Gammaproteobacteria, P.adjust = 0.05, fc = 136.14) and *Parasutterella* (Betaproteobacteria, P.adjust = 0.04, fc = 1.97) were reduced as compared to baseline.

The only difference between the regular rye bread versus baseline samples was the reduction of *Flavonifractor* (*Ruminococcaceae*) (P.adjust = 0.01, fc = 14.83). Although not reaching significance after adjustment for multiple comparisons, there was a tendency towards an increase in the abundance of bifidobacteria during the low-FODMAP rye bread period (*P* = 0.03, P.adjust = 0.22, fc = 1.42), paralleled with a weaker upward trend during the regular rye bread period (*P* = 0.28, P.adjust = 0.70, fc = 1.20). The interventions had no effects on other bacteria including *Faecalibacterium prausnitzii* and *Lactobacillus spp*. that have previously been associated with consumption of a low-FODMAP diet.

### Associations of the microbiota to IBS symptoms and to the individual variation in bread intake-related symptom control

Correlation analysis between the microbiota and IBS symptoms (IBS-SSS, visual analogue scale (VAS) assessments of individual symptoms and H2 production) during the intervention yielded only two significant associations: H2 production was positively associated with the abundance of *Anaerostipes* (r = 0.31, *P* = 0.003), and a weak positive correlation was observed between constipation and *Clostridia* FamilyXIII Incertae Sedis (r = 0.17, *P* = 0.035). As both the intestinal microbiota and the tolerance to FODMAPs varied considerably among IBS patients, it was thought that subgroup analyses could provide novel insights into the relationship between the microbiota and symptoms. Hence, we zoomed in on those patients who experienced the most intense change in symptom control during the interventions and asked if patients who enjoyed symptom relief (responders) after consumption of the low-FODMAP bread had a different microbiota composition compared to the rest, here classified as non-responders.

Based on the criteria described in the methods, we identified 18 responders and 31 non-responders for the low-FODMAP bread. The individual GI symptoms that differed significantly between the groups were flatulence (*P* = 0.004), dyspepsia (*P* = 0.005) and heartburn (*P* = 0.04); these symptoms were all significantly decreased in the responders when they were consuming the low-FODMAP bread (Additional file 3: Figure S3A-C). The amount of breath hydrogen (H_2_) was also significantly elevated in the non-responders (*P* = 0.01, Additional file 3: Figure S3D). The dietary intakes did not differ between the responder groups at baseline but during the low-FODMAP rye bread period, the non-responders consumed significantly less fibre (24.3 g vs. 30.1 g, P = 0.04). The responder status was not affected by IBS-subgrouping (IBS-D, IBS-M or IBS-U), gender or age.

**Fig. 3:**
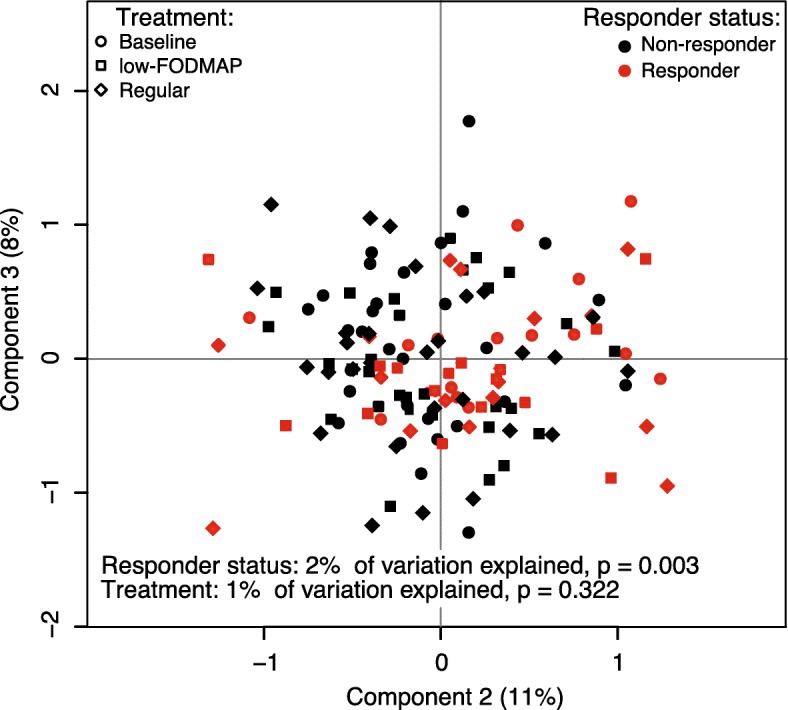
Principal coordinates analysis (PCoA) plot of genus-level data based on Bray-Curtis dissimilarity. Samples are colored according to responder status, defined by lower scores of IBS-SSS and/or pain during the low FODMAP rye bread period compared to baseline. The symbols depicting the treatment. Percentage of the total microbiota variation explained by both variables and their *p*-values were calculated with permutational multivariate ANOVA

**Fig. 4:**
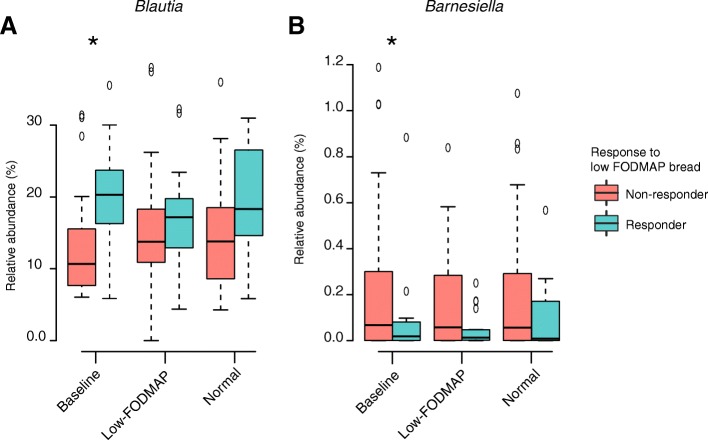
Relative abundance of bacterial genera that differed significantly between the responders and non-responders; responders were identified by lower scores of IBS-SSS and/or pain during the low FODMAP rye bread period compared to baseline. The box extends from 25th percentile to 75th percentile, with a line at the median; the whiskers refer to the highest and lowest values. Abundances are plotted for baseline and for both bread periods and those with a statistically significant (adjusted *p* < 0.05) difference are indicated with an asterisk

Considering the overall microbiota, a higher fraction of the variation was attributable to the low-FODMAP bread-related responder status (2%, *P* = 0.003) than to the intervention itself (1%, *P* = 0.3; Fig. 3). The microbial α-diversity was not affected by the responder status. At baseline, the amount of *Blautia* was increased in the responders (P.adjust = 0.01, fc = 1.6) whereas those of *Barnesiella* (*Porphyromonadaceae*, Bacteroidales) were reduced in the responders (P.adjust = 0.03, fc = 0.22, Fig. 4). Finally, as a reference, the microbiota between the subjects whose symptoms were strongly triggered after the intake of the regular rye bread versus were compared to those without a major symptom change. Here, we identified 22 responders and 28 non-responders, with no microbiota differences between the groups (for all taxa P.adjust > 0.05).

## Discussion

This is the first time that the effects of regular and low-FODMAP rye bread on the intestinal microbiota of IBS patients have been investigated. The comparison of two active treatments is usually conceived as the key analysis in a cross-over trial. However, we did not observe any differences between the breads in terms of the microbiota, apart from a presumably anecdotal finding on the difference abundance of *Klebsiella.* Instead, our results indicate that both breads induced parallel but non-identical changes in the microbiota as compared to baseline, which is logical as the increase in the daily fibre intake of 7–8 g/d is likely to be a stronger stimulus to the microbiota compared to the about 1 g difference in the fructan intakes between the two bread periods.

Compared to baseline, consumption of the low-FODMAP rye bread led to detectable but a non-significant increase of bifidobacteria as well as decreases in several of the genera (Fig. 2) that can be considered as potentially harmful for IBS patients (see below). Our findings are promising since the reduction in the numbers of bifidobacteria has been recognized as a dysbiotic finding in IBS patients [21] and an adverse effect of the low-FODMAP diet. These and our previous results [12] indicate that low-FODMAP rye bread represents an innovative component of an IBS diet as it improves some symptoms more than the regular rye bread, substantially increases fibre intake and x consequently results in apparently beneficial microbial changes in the gut.

Our study adds to the previous evidence that the addition or substitution of specific, high-fibre grains/cereals while the individual continues with his/her otherwise habitual diet does not alter the microbial diversity or cause other community-wide microbiota effects [22–25]. Instead, specific changes in individual bacterial taxa were observed when comparing the baseline versus post-intervention microbiota (Fig. 2). Recently, Swedish researchers studied the effects of refined wheat, whole grains wheat and rye in 70 healthy volunteers [23]. The intestinal microbiota composition was not affected by any of these grain products in that 6-week cross-over study. Similarly, a parallel-arm intervention trial conducted in Finnish metabolic syndrome patients consuming a diet with high amounts of rye at baseline, did not change the microbiota composition between the subjects who consumed either white wheat or rye bread for 12 weeks [24]. In our study, the intake of rye and other grains was relatively low in the habitual diet, and hence the introduction of the study rye breads substantially altered the intake of high fibre grains.

The low-FODMAP rye bread decreased the relative amounts of *Bacteroides, Flavonifractor, Holdemania, Klebsiella and Parasutterella* (Fig. 2) as compared to baseline. Members of the genus Bacteroides are abundant commensals but some species are also significant clinical pathogens [26], therefore the abundance changes of this group cannot be regarded as either beneficial or detrimental for IBS patients. Inhabitants of Western countries typically have a very high abundance of *Bacteroides* as compared to those living in Africa where the diet is heavily plant-based and contains high amounts of fibre [27]. In this trial, the amounts of *Flavonifractor* were reduced during both intervention periods when compared to baseline. This organism uses gamma-aminobutyric acid (GABA) as a growth substrate, and hence presumably reduces the amount of GABA in the gut [28]. As GABA has multiple regulatory effects in the intestine including a reduction of both transit time and pain [29], our finding that an increased intake of cereal fiber leads to a reduction in the numbers of “GABA-eating” Flavonifractor in IBS patients is rather interesting. Recently, elevated levels of *Flavonifractor* were found in the rectal mucosa of autistic children with functional gastrointestinal disorders, especially in those individuals reporting abdominal pain [30]. While GABA was not measured in that study, the amount of *Flavonifractor* correlated linearly with the levels of serotonin in the tissue biopsy specimens. Increased levels of *Flavonifractor* have also been assayed in feces of depressed adults [31], further strengthening the rationale for additional studies to clarify the link between *Flavonifractor*, intestinal pain and mood disorders. Furthermore, GABA analogs such as gabapentin and pregabalin have been reported to reduce visceral hypersensitivity in preclinical models [32] and pregabalin has shown promise in reducing abdominal pain in a clinical trial with IBS-patients [33]. In addition, an increased abundance of bifidobacteria, i.e. as occurred during consumption of the low-FODMAP rye bread, has been reported to associate with a reduced level of abdominal pain in cross-sectional studies of IBS patients [34–36]. Taken together, these findings suggest that dietary modifications that decrease the abundance of *Flavonifractor* and increase the abundance of bifidobacteria might reduce the abdominal pain or accelerated transit time in IBS.

Similarly to *Flavonifractor, Parasutterella,* the most abundant proteobacterium in autistic children [37], has been associated with abdominal pain in autistic children [30]. *Klebsiella* is an opportunistic pathogen that is a common cause of urinary tract infections and capable of acquiring resistance to antibiotics. Its abundance has been claimed to be high in some IBS subjects [38]. *Holdemania* has been reported as a key member of bacterial networks of pediatric IBS patients [39] and to be enriched in gout patients as compared to healthy controls [40]. While the clinical implications of our findings of the low-FODMAP diet induced microbiota alterations remain unclear, it is evident that any reduction in the numbers of the above-mentioned bacteria are likely to have beneficial rather than harmful effects in IBS patients.

Our results suggest that prebiotic oligosaccharides in grains belonging to the FODMAP family, such as fructans, can be substituted with other grain-based prebiotic factors to improve the abundance of bifidobacteria and to reduce IBS symptoms [12]. Previous in vitro studies have shown that arabinoxylan-fibre from rye stimulates the growth of human bifidobacteria [41, 42]. A randomised trial in healthy volunteers revealed that arabinoxylan enriched rye/wheat products increased the fecal butyrate concentration and tended to increase the abundance of bifidobacteria [22]. However, more studies will be needed to clarify the clinical significance of the increase in the abundance of bifidobacteria in IBS patients. In addition, it would be interesting to investigate the effects of non-FODMAP fibre components, especially arabinoxylan, on the production of short-chain fatty acids and on host metabolomics. Ultimately, only prospective long-term studies will demonstrate if the increase in the abundance of bifidobacteria will translate into beneficial clinical outcomes in IBS patients.

The stratification of the subjects according to their response to the low-FODMAP bread intake based on the IBS-SSS and pain scores revealed that those patients who benefitted from the intake of low-FODMAP rye bread (responders) had a different microbiota composition at baseline than those whose symptoms were not improved (non-responders). In particular, *Blautia,* a bacterial species that decreases intestinal gas by producing acetate [42], was significantly more abundant in responders versus non-responders (mean 19% vs 14%, respectively). Previously, Chumpitazi et al. have demonstrated that pediatric IBS patients whose symptoms markedly improved while adhering to a low-FODMAP diet had a different microbiota composition as compared to those who did not exhibit any improvement [14, 15]. In addition, adult IBS patient responders to low-FODMAP diet have been previously discriminated from non-responders before and after intervention based on their fecal bacterial profiles [6]. In our study, the subjects benefitting from the low-FODMAP rye bread had significantly more *Blautia* at baseline, suggesting that the ability to remove hydrogen might be at least as important a determinant of IBS symptom control than the generation of intestinal gas. *Blautia* utilizes hydrogen and carbon dioxide to form acetate [43] and since it is one of the most dominant intestinal genera, it presumably exerts a significant role in reducing the intestinal gas volume and pressure. These data suggest that increasing the abundance of Blautia might have potential as a new therapeutic target in IBS. The other bacterium that differed in abundance between the responder groups was *Barnesiella,* which was lower in responders. In a recent meta-analysis, *Barnesiella* was shown to be more abundant in control subjects as compared to those with intestinal diseases [44]. We did not detect any bacteria to be specifically associated with a worsening of IBS symptoms following the intake of regular rye bread. This may reflect the fact that many different bacteria are involved in the fermentation of dietary fibre and hence the main gas-producing bacteria that contribute to the symptoms probably vary between different individuals and cannot be quantified with phylogenetic microbiota analysis.

One limitation of our study is that we did not include patients with constipation predominant-IBS (IBS-C), as we wished to keep the patient cohort as homogenous as possible. Therefore, the results are not directly transferable to patients with IBS-C. There is no database in Finland on the FODMAP content of foods, especially data on fructans and polyols is lacking; thus we were unable to calculate the total amount of FODMAPs consumed during the periods. Some participants may have either reduced or increased their intake of other FODMAPs accidently or intentionally during the study despite our instructions. It is noteworthy that the intake of fibre increased substantially during both intervention periods; and only a difference of 1 g was noted owing to different fructan content of the breads. We did not analyse markers of low-grade inflammation and intestinal permeability but a recent study indicated that a high intake of rye may reduce low-grade inflammation [24]. The measurements of these markers might have shed light on the clinical relevance of the observed microbial changes. Two studies investigating the effects of a low-FODMAP diet have shown that even although some changes in the microbiota appear to be unfavourable, the overall effects of the low-FODMAP diet on low-grade inflammation and immune activation seem to be beneficial [7, 45]. Therefore, it is premature to conclude if the microbial changes observed in IBS or during different dietary interventions will translate into clinically meaningful outcomes.

The strength of our study is its double-blinded setting, which is not often possible in dietary interventions. Furthermore, the observed beneficial effects in the microbiota were achieved by consuming a staple food, i.e. bread. People with IBS may find it easier to adhere to food modification in the long-term in comparison with consuming probiotic or other relatively expensive dietary supplements that might also increase the abundance of at least some bifidobacteria strains among people with IBS [46].

## Conclusions

In conclusion, our double-blind study demonstrated that consumption of a high-fibre low-FODMAP rye bread displayed the potential to support healthy microbiota. This kind of low-FODMAP rye bread might be a practical means of improving long term overall gut health in IBS patients. Our results also can act as a foundation for further studies focusing on gut microbiota and individual responses to clarify the mechanisms underpinning diet-induced gastrointestinal symptoms as well as targeting dietary therapy to those individuals in whom they will exert the maximum clinical benefits.

## Additional files

Additional file 1: **Figure S1.** Patient flow. (PDF 102 kb)

Additional file 2: **Figure S2.** Principal coordinates analysis (PCoA) plot of genus-level data based on Bray-Curtis dissimilarity. Samples are colored according to intervention order symbols depicting the treatment. Percentage of the total microbiota variation explained by the treatment and the *p*-value were calculated with permutational multivariate ANOVA. (PDF 114 kb)

Additional file 3: **Figure S3.** a-c) Individual symptoms for which the scores differed significantly (*p* < 0.05) between the IBS-SSS and pain-defined responders and non–responders during the low-FODMAP rye bread consumption. d) Differences in hydrogen excretion between the IBS-SSS and pain-defined responders. Statistically significant differences are indicated with an asterisk. (PDF 130 kb)

## Abbreviations

16S rRNA: 16 S Ribosomal Ribonucleic Acid
ANOVA: Analysis of variance
DNA: Deoxy ribonucleic acid
FODMAP: Fermentable, Oligo-, Di-, Mono-saccharides and Polyols
GABA: Gamma-aminobutyric acid
HMWDF: High Molecular Weight Dietary Fibre
IBS: Irritable Bowel Syndrome
IBS-D: Diarrhoeal predominant subgroup
IBS-M: Mixed subgroup of IBS
IBS-SSS: IBS Symptom Severity Score
IBS-U: Unspecified subgroup of IBS
LMWDF: Low Molecular Weight Dietary Fibre
RBB: Repeated Bead Beating
