# Supplementary figures and images for: Randomised clinical trial: effect of low-FODMAP rye bread versus regular rye bread on the intestinal microbiota of irritable bowel syndrome patients: association with individual symptom variation
Source: BMC Nutr. 2019 Mar 6;5:12. doi: 10.1186/s40795-019-0278-7 (PMC7050854; doi:10.1186/s40795-019-0278-7)

## Order of study breads

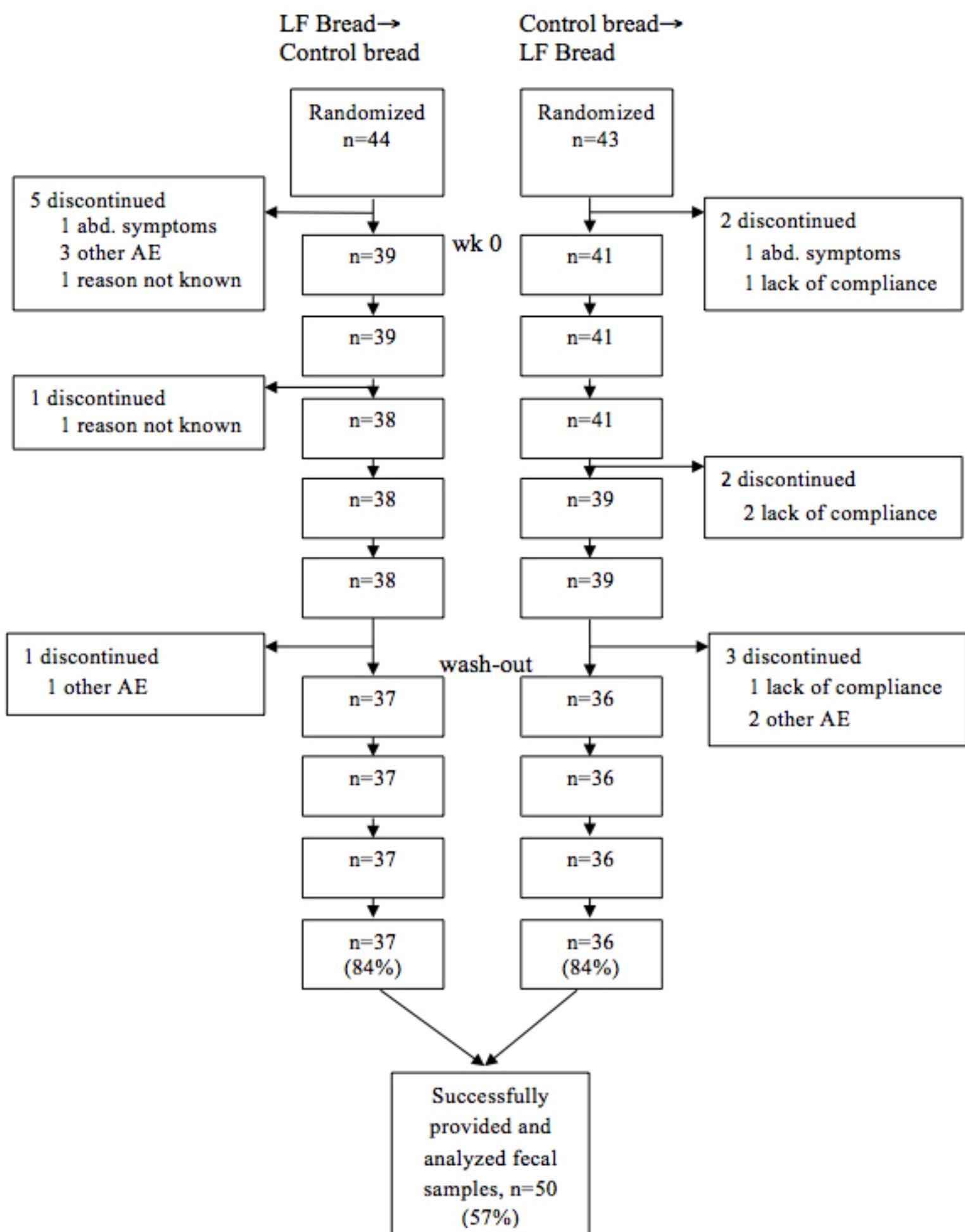

Supplement: Supplementary file 1 — Figure S1. Patient flow. (PDF 102 kb) [file 40795_2019_278_MOESM1_ESM.pdf]

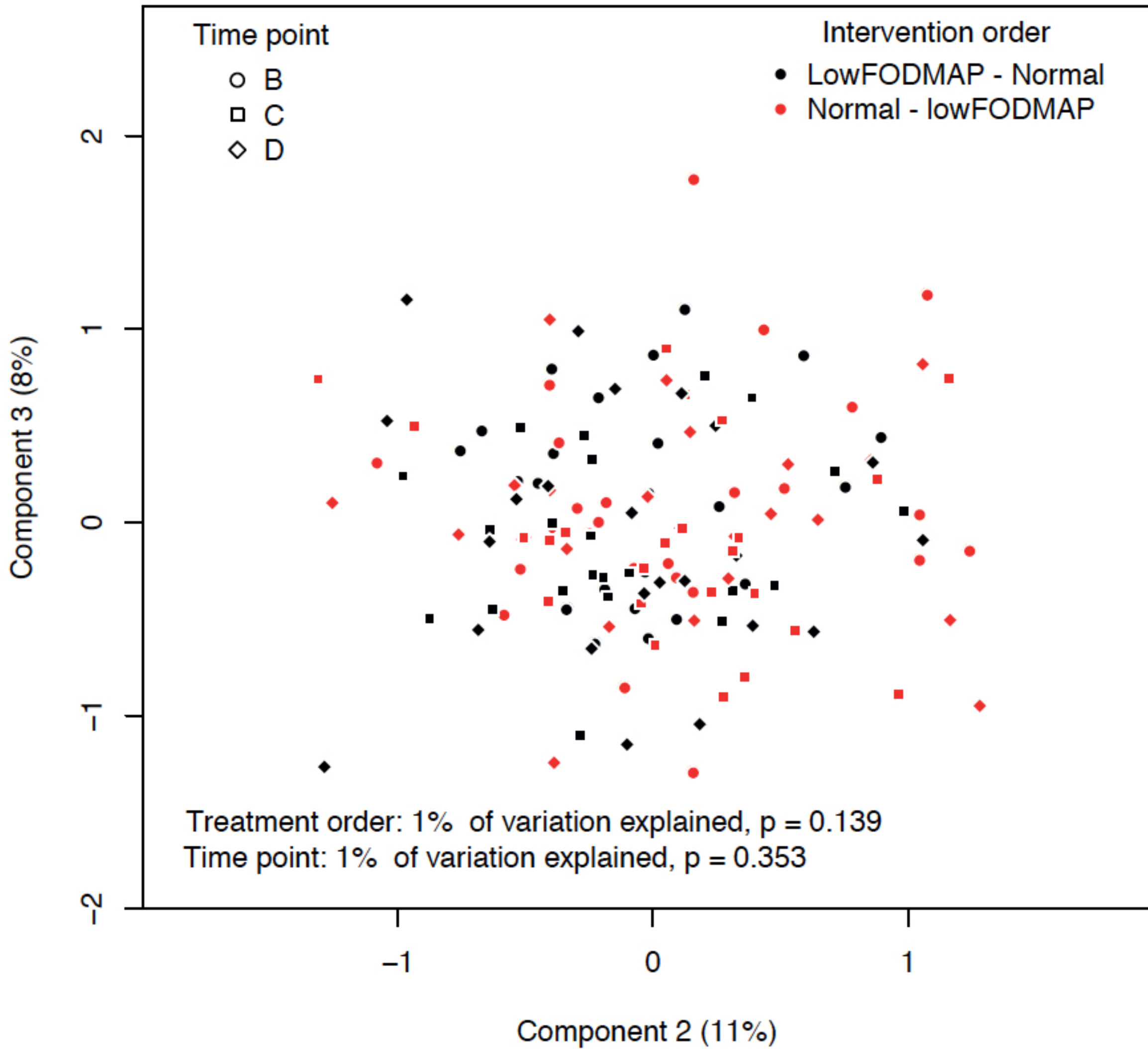

Supplement: Supplementary file 2 — Figure S2. Principal coordinates analysis (PCoA) plot of genus-level data based on Bray-Curtis dissimilarity. Samples are colored according to intervention order symbols depicting the treatment. Percentage of the total microbiota variation explained by the treatment and the p-value were calculated with permutational multivariate ANOVA. (PDF 114 kb) [file 40795_2019_278_MOESM2_ESM.pdf]

A

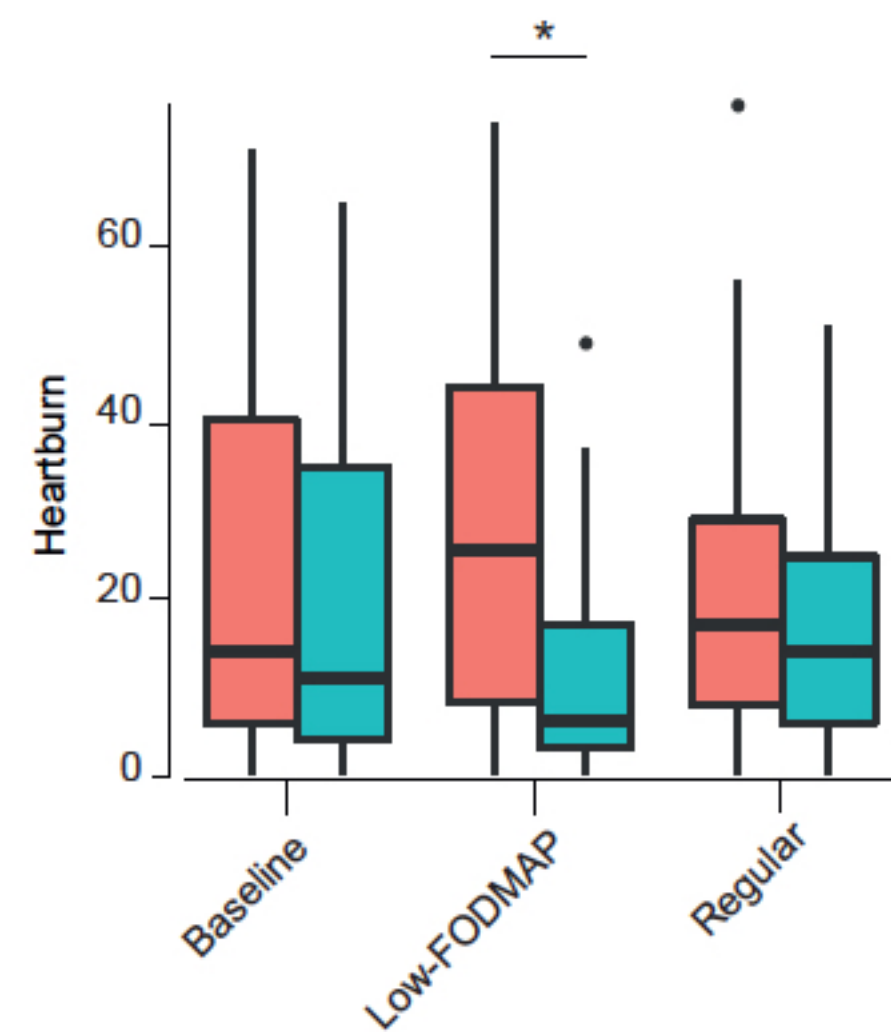

B

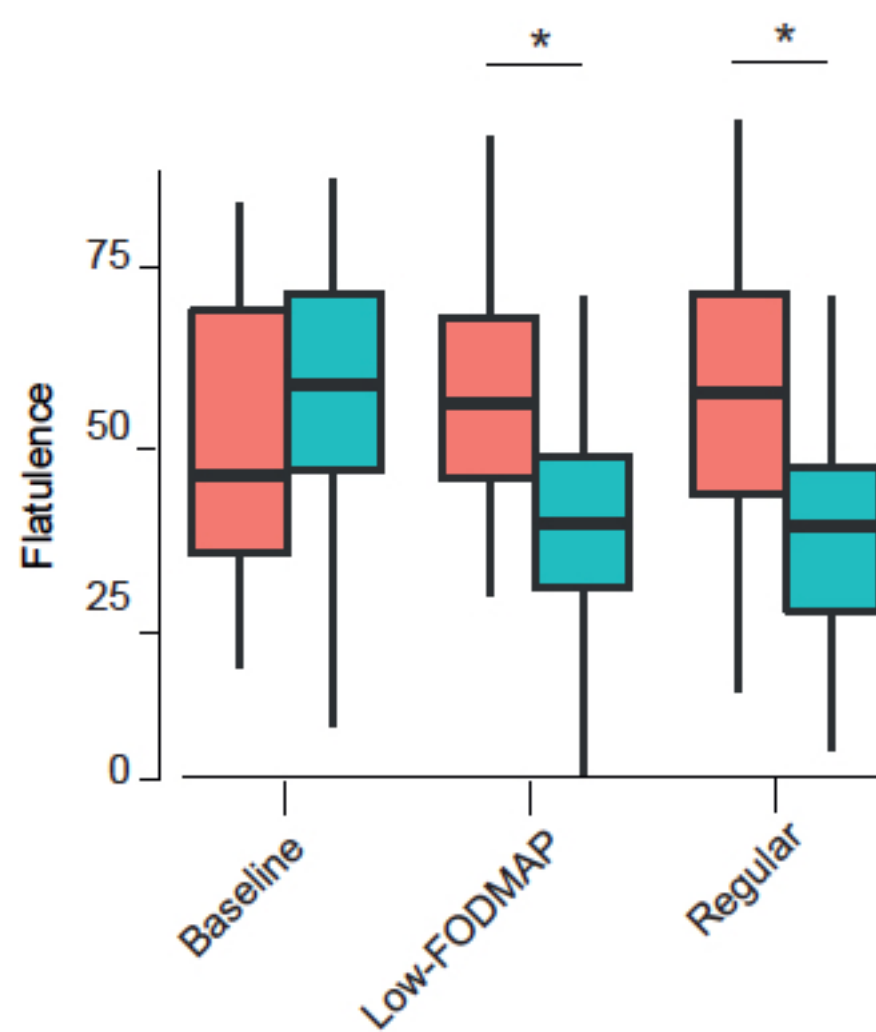

C

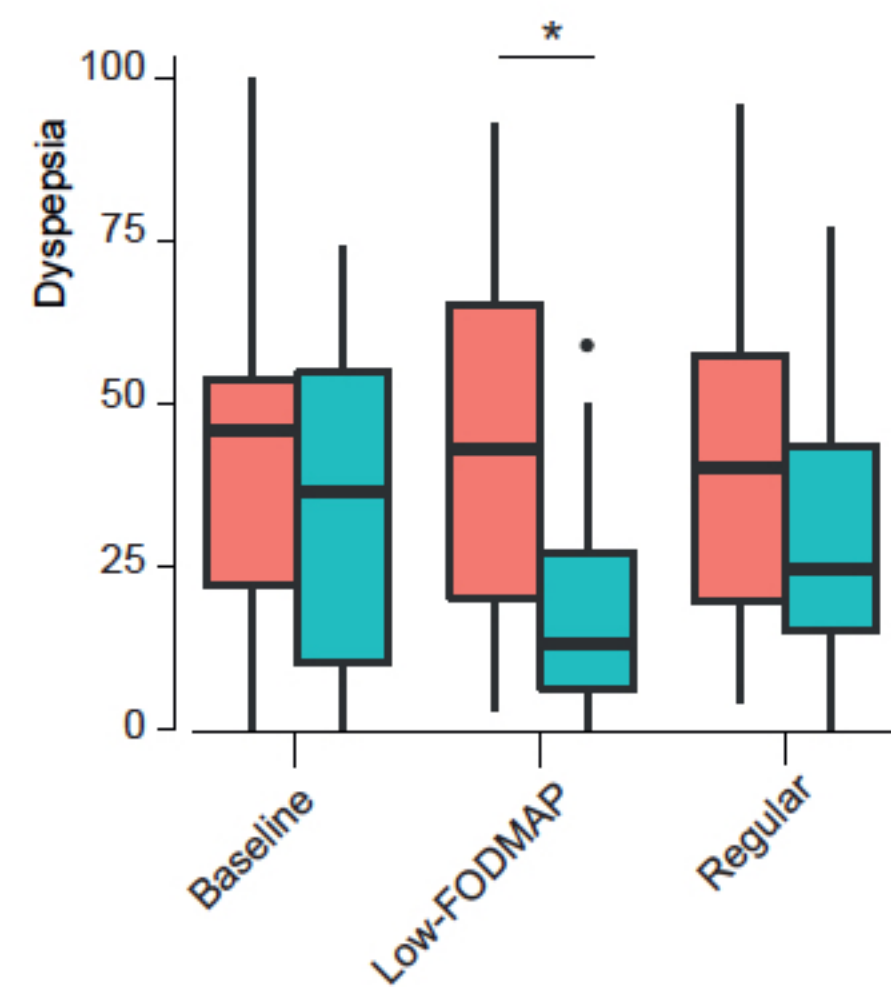

D

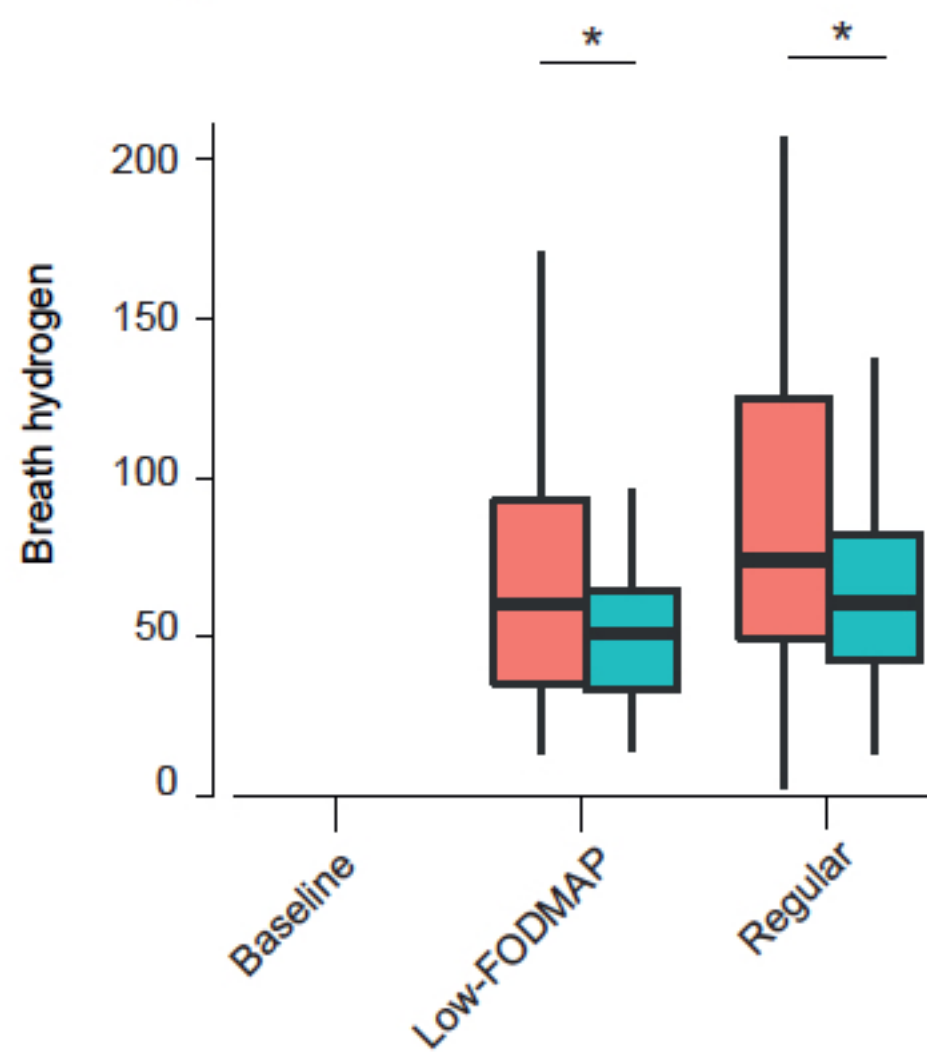

Response to  
low FODMAP bread

Non-responder

Responder

Supplement: Supplementary file 3 — Figure S3. a-c) Individual symptoms for which the scores differed significantly (p < 0.05) between the IBS-SSS and pain-defined responders and non–responders during the low-FODMAP rye bread consumption. d) Differences in hydrogen excretion between the IBS-SSS and pain-defined responders. Statistically significant differences are indicated with an asterisk. (PDF 130 kb) [file 40795_2019_278_MOESM3_ESM.pdf]
